# Cytotaxonomy of *Gallinula melanops* (Gruiformes, Rallidae): Karyotype evolution and phylogenetic inference

**DOI:** 10.1590/1678-4685-GMB-2020-0241

**Published:** 2021-04-02

**Authors:** Ivanete de Oliveira Furo, Rafael Kretschmer, Patricia C. M. O’Brien, Jorge Claudio da Costa Pereira, Ricardo José Gunski, Analía Del Valle Garnero, Rebecca E. O’Connor, Darren Karl Griffin, Malcolm A. Ferguson-Smith, Edivaldo Herculano Corrêa de Oliveira

**Affiliations:** 1 Universidade Federal Rural da Amazônia (UFRA) Laboratório de Reprodução Animal (LABRAC), Parauapebas, PA, Brazil.; 2 University of Cambridge Department of Veterinary Medicine, Cambridge Resource Centre for Comparative Genomics, Cambridge, United Kingdom.; 3 Universidade Federal do Rio Grande do Sul, Programa de Pós-graduação em Genética e Biologia Molecular (PPGBM), Porto Alegre, RS, Brazil.; 4 University of Kent, School of Biosciences, Canterbury, United Kingdom.; 5 University of Trás-os-Montes and Alto Douro (UTAD), Animal and Veterinary Research Centre (CECAV), Vila Real, Portugal.; 6 Universidade Federal do Pampa, Programa de Pós-graduação em Ciências Biológicas (PPGCB), São Gabriel, RS, Brazil.; 7 Instituto Evandro Chagas, Laboratório de Cultura de Tecidos e Citogenética (SAMAM), Ananindeua, PA, Brazil.; 8 Universidade Federal do Pará, Instituto de Ciências Exatas e Naturais, Belém, PA, Brazil.

**Keywords:** Birds, Clade Fulica, chromosome painting, chromosome evolution, microchromosomes

## Abstract

Although Rallidae is the most diverse family within Gruiformes, there is little information concerning the karyotype of the species in this group. In fact, *Gallinula melanops*, a species of Rallidae found in Brazil, is among the few species studied cytogenetically, but only with conventional staining and repetitive DNA mapping, showing 2n=80. Thus, in order to understand the karyotypic evolution and phylogeny of this group, the present study aimed to analyze the karyotype of *G. melanops* by classical and molecular cytogenetics, comparing the results with other species of Gruiformes. The results show that *G. melanops* has the same chromosome rearrangements as described in *Gallinula chloropus* (Clade Fulica), including fission of ancestral chromosomes 4 and 5 of *Gallus gallus* (GGA), beyond the fusion between two of segments resultants of the GGA4/GGA5, also fusions between the chromosomes GGA6/GGA7. Thus, despite the fact that some authors have suggested the inclusion of *G. melanops* in genus *Porphyriops*, our molecular cytogenetic results confirm its place in the *Gallinula* genus.

## Introduction

Gruiformes is an avian order showing great heterogeneity of habits, habitats and morphology and a wide geographic distribution (Del Hoyo et al., 1996; Garcia et al., 2014). Because of their great diversity, phylogenetic relationships among the different families in this order are still controversial, despite the number of phylogenetic studies performed so far. One of the first proposals based on morphological characters classified Gruiformes into 12 families (Wetmore, 1960). However, with the introduction of new methods, such as molecular studies using mitochondrial-nuclear DNA or genome sequencing, it has been possible to reach a consensus that there is a monophyletic core of five families known as “Core Gruiformes”:Rallidae, Heliornithidae, Psophiidae, Aramidae, and Gruidae (Fain et al., 2007; Hackett et al., 2008; Jarvis et al., 2014; Prum et al., 2015).

Within the core Gruiformes, Rallidae is the family with the highest number of species, around 152, distributed in 33 to 40 genera, comprising 85% of the order diversity (Garcia et al., 2014; Gill et al., 2020). The phylogenetic relationships within Rallidae still present many inconsistencies, due mainly to the small numbers of species that have been sampled in the different approaches (Garcia *et al*., 2014).

Rallidae shows a huge geographic distribution and taxonomic complexity (Livezey, 1998). Several species of this family, including the Spot-flanked Gallinule (*Gallinula melanops*), are distributed from northeastern to southern Brazil, with occurrences in Bolivia, Paraguay and Argentina southward (Taylor, 1996; Taylor and van Perlo, 1998). *G. melanops* (2n=80) is one of the six Rallidae species with known karyotypes, together with *Fulica atra* (2n=92), *Gallinula chloropus* (2n=78), *Aramides cajaneus* (2n=78), *Porzana albicollis* (2n=72), and *Porphyrio porphyrio* (2n=80) (Giannoni and Giannoni, 1983; Hassan, 1998; Nanda et al., 2011; Gunski et al., 2019; Furo et al., 2020). Hence, in the classification proposed by Garcia et al. (2014), only species belonging to clade Fulica (genera *Fulica*, *Gallinula* and *Porzana*), Aramides (*Aramides* and *Porzana albicollis*) and Porphyrio (genus *Porphyrio*) have been analyzed by classical or molecular cytogenetics.

The introduction of new cytogenetic tools, especially comparative chromosome painting has helped to improve the understanding of karyotype evolution and phylogenetic relationships among different species of birds (Kretschmer et al., 2014, 2015, 2018a, 2020; Furo et al., 2015, 2018; Rodrigues et al., 2018). The variety of whole chromosome painting probes now available include chicken (*Gallus gallus*-GGA), stone-curlew (*Burhinus oedicnemus*-BOE), white hawk (*Leucopternis albicollis*-LAL), griffon vulture (*Gyps fulvus*-GFU) and eared dove (*Zenaida auriculata*-ZAU) (Nie et al., 2009; de Oliveira et al., 2010; Kretschmer *et al*., 2018b). Moreover, the use of bacterial artificial chromosomes (BACs) from the genome library of *G. gallus* has overcome much of the difficulty in the analysis of microchromosome rearrangements (Lithgow et al., 2014; O’Connor et al., 2019).

There is an urgent need to use these new techniques to clarify the problems concerning avian karyotypes and phylogenetic relationships in a greater number of species (Dobigny et al., 2004; Furo et al., 2015, 2018; Nie et al., 2015; Seligmann et al., 2019). The main aim of this study was to characterize the karyotype of *G. melanops* by classic cytogenetics, GGA chromosome painting probes and FISH with BACs selected from the genome library from microchromosomes of *G. gallus* in order to contribute to the phylogeny and karyotype evolution of the Rallidae family.

## Material and Methods

### Chromosome preparation

Fibroblast cultures obtained from wing skin biopsies of five female specimens of *Gallinula melanops* were collected in São Gabriel, Rio Grande do Sul State, (RS, Brazil), following Sasaki et al. (1968) with modifications. The samples were first mechanically fractionated in a Petri dish after incubation in type IV collagenase for tissue dissociation. The cells were cultured in DMEM (GIBCO) supplement with calf bovine serum 20%, Aminiomax^TM^ -II 5% and penicillin (PNS) 1% then incubated at 37 ºC. Afterwards, metaphase arrest was obtained by adding colcemid (Gibco, 100 μl for 5 ml of complete medium) followed by incubation for 1 hour at 37 ºC, and hypotonic solution treatment (KCl 0,075 M) for 15 minutes. Finally, the suspensions were fixed using Carnoy’s fixative methanol: acetic acid (3:1 v/v). The experiments followed ethical protocols approved by the Ethics Committee nº018/2014 (UNIPAMPA) and SISBIO: 44173-3.

### **Fluorescence *in situ* Hybridization (FISH)**

*Gallus gallus* (GGA) chromosome probes from 1 to 14 obtained by flow-sorting and labeled with biotin-16-dUTP or digoxigenin-11-dUTP (Roche Diagnostics, Mannheim, Germany) by degenerate oligonucleotide-primed polymerase chain reaction (DOP-PCR) (Telenius et al., 1992) were hybridized to metaphase chromosomes of *G. melanops*, following standard protocols, as described previously by de Oliveira et al. (2010). In this study ZW chromosome probes of *G. gallus* were not used. The FISH results were analyzed using a Zeiss Imager 2 microscope, 63x objective and images were captured using Axiovision 4.8 software (Zeiss, Germany). At least 10 metaphases were analyzed to confirm the hybridizations signals. Final editing of images was performed using Adobe Photoshop CC software. For chromosomal evolution inferences, we used chromosome painting data from *Fulica atra*, *Gallinula chloropus* and *Aramides cajaneus* (Nanda et al., 2011; Furo et al., 2020), also these data were plotted in a phylogenetic tree proposed by Garcia et al., (2014), to clarify the phylogenetic position of some Rallidae species.

The bacterial artificial chromosomes (BACs) were selected from the genome library of *G. gallus* or *Taeniopygia guttata* (Zebra finch) for the microchromosomes GGA17-28, following O’Connor et al. (2019). Slides were analyzed with an Olympus BX-61 epifluorescence microscope equipped with a cooled CCD camera and appropriate filters. Images were captured using SmartCapture3 (Digital scientific UK).

## Results

### **Karyotype and chromosome painting with *G. gallus* probes**

**Figure 1 -:**
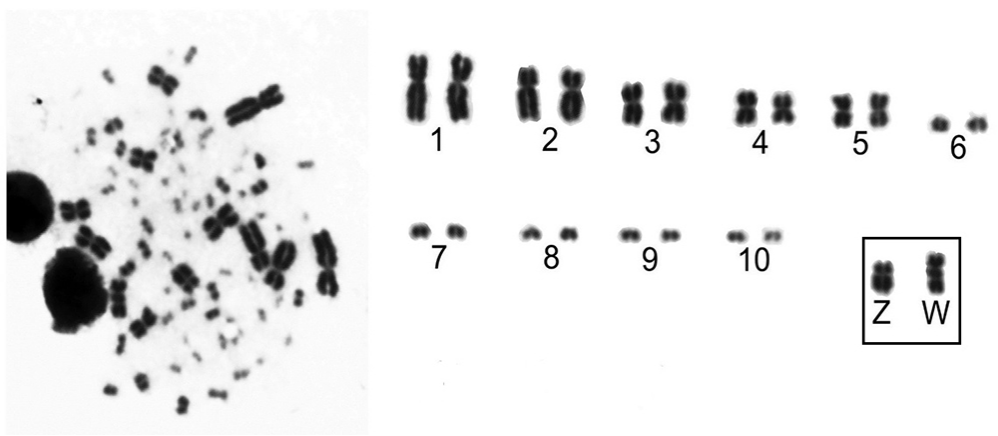
Partial karyotype of *Gallinula melanops* (2n=80), stained with Giemsa showing only the macrochromosomes 1-10 and sex chromosomes ZW.

The karyotype of *Gallinula melanops* (2n=80) is composed of 11 pairs of macrochromosomes, including the Z and W, and 29 pairs of microchromosomes, which corroborates previous findings (Gunski et al., 2019). The first and second chromosome pairs are submetacentrics, the third, fourth and fifth pairs are metacentrics and the other chromosome are telocentrics. The sex chromosomes ZW are submetacentrics, and the W is larger than the Z chromosome due to the accumulation of repetitive DNA, as described by Gunski *et al*. (2019) (Figure 1).

**Figure 2 -:**
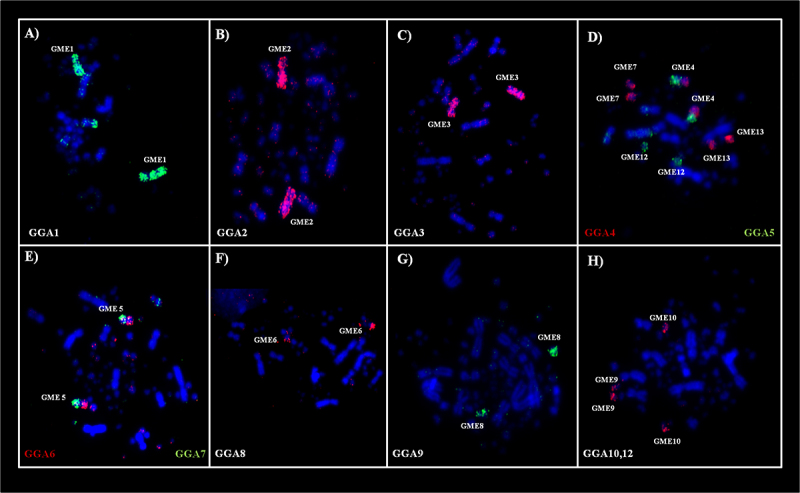
FISH using whole-chromosome *Gallus gallus* probes on metaphases of *Gallinula melanops*. A-C and F-H are examples of conserved chromosomes, while D-E are examples of fusions and fissions. (D) GME4=GGA4 + GGA5 => fusion; GGA4 = GME4p + GME7+ GME13 => fission; GGA5 = GME12 + GME4q => fission; and (E) evidence that GGA6 + GGA7 = GME 5 => fusion.

**Figure 3 -:**
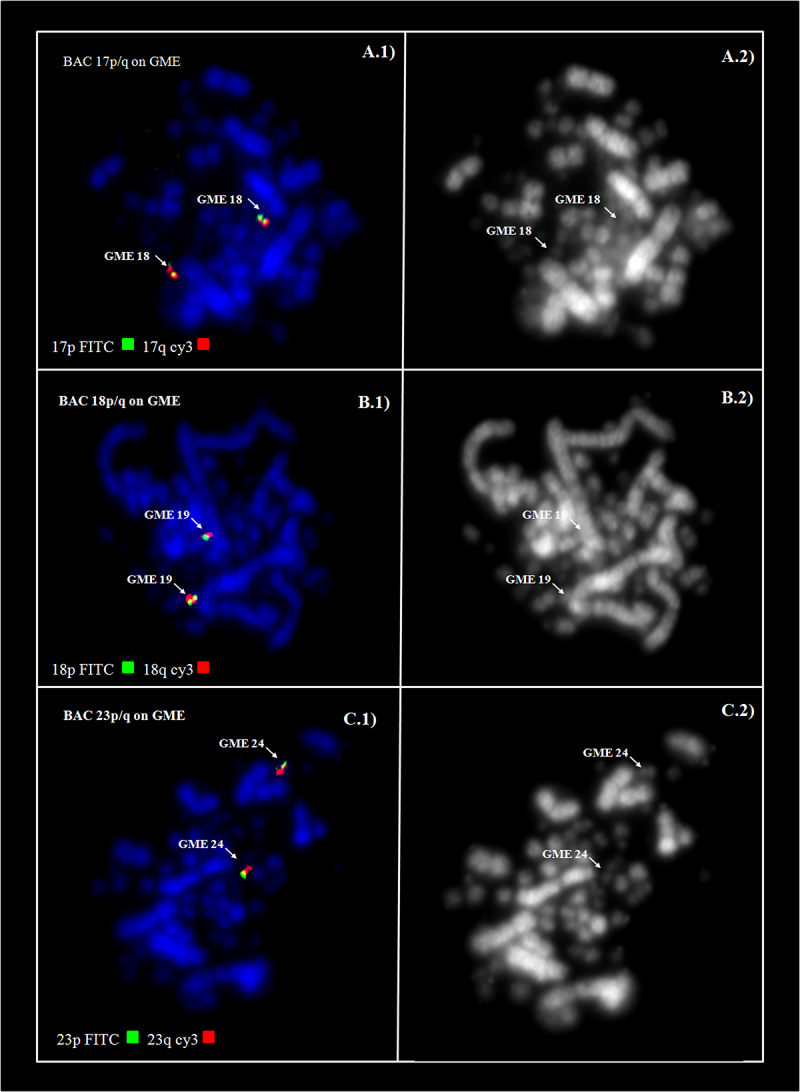
Homology between chromosomes of *Gallus gallus* (GGA) and *Gallinula melanops* (GME) determined by FISH experiments using Chicken BACs from microchromosomes. Examples of conserved microchromosomes can be observed in: A1-A2- BACs 17p/q, B1-B2- BACs 18p/q, C1-C2- BACs 23p/q.

**Figure 4 -:**
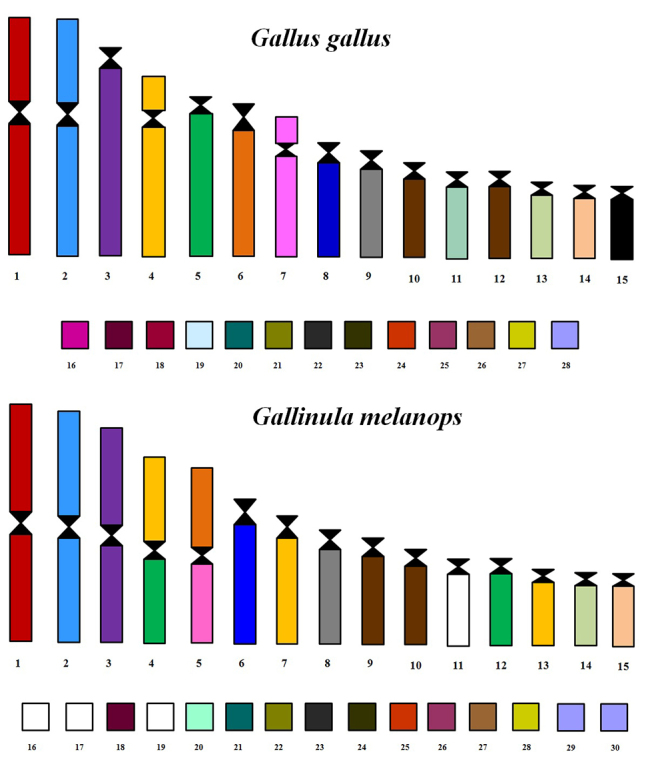
Homology map between chromosomes of *Gallus gallus* and *Gallinula melanops* determined by FISH experiments with GGA painting probes (GGA1-14) and GGA BAC clones of microchromosomes from 17-28. A) reporting the colour guide to GGA painting probes and BAC clones, and B) reporting the homology between the chromosomes of these two species. Segments not hybridized are indicated in white. Chicken probe 11 did not work and BACs 15 and 16 were not used in this study. The BAC 20 of Zebra finch is represented by the BAC 20 of chicken.

The hybridization of fluorescent whole chromosome probes from *G. gallus* to the metaphase chromosomes of *G. melanops* shows fission events involving of ancestral cromosomes GGA4 and GGA5, beyond the fusion between two of segments resultants of the GGA4 and GGA5 (Figure 2D). Also, fusions were observed between chromosomes GGA6 and GGA7. The chromosome correspondences were: GGA1- GME1; GGA2- GME2; GGA3- GME3; GGA4- GME4p, GME7 and GME13; GGA5- GME4q and GME12; GGA6- GME5p; GGA7- GME5q; GGA8- GME6; GGA9- GME8; GGA10-12- GME9 and GME10; GGA13- GME14; GGA14- GME15 (Table 1 and Figures 2 and 4). Each chicken or Zebra finch BAC probe from microchromosomes 17 to 28 hybridized only to one pair of microchromosomes, revealing that they were conserved and did not participate in interchromosomal rearrangements (Table 2, Figure 3).

**Table 1 -:** Correspondence between *Gallus gallus* (GGA) and *Gallinula melanops* (GME) chromosomes.

Probes	Pair number	*G.melanops*
GGA1	1 pair	GME1
GGA2	1 pair	GME2
GGA3	1 pair	GME3
GGA4	3 pair	GME 4p, 7, 13
GGA5	2 pair	GME 4q, 12
GGA6	1 pair	GME 5p
GGA7	1 pair	GME 5q
GGA8	1 pair	GME 6
GGA9	1 pair	GME 8
GGA10-12	2 pairs	GME 9, 10
GGA11	-	-
GGA13	1 pair	GME 14
GGA14	1 pair	GME 15

**Table 2 -:** Correspondence between *Gallus gallus* BACs from microchromosome and *Gallinula melanops* microchromosomes.

BAC names	*G.gallus*	*G.melanops*
CH261-42P16	17q	18q
CH261-113A7	17p	18p
CH261-72B18	18q	19q
CH261-60N6	18p	19p
CH261-10F1	19q	20q
CH261-50H12	19p	20p
TGMCBA-250E3	20q	21q
TGMCBA-341F20	20p	21p
CH261-122K8	21q	22p
CH261-83I20	21p	22q
CH261-18G17	22q	23q
CH261-40J9	22p	23p
CH261-90K11	23q	24q
CH261-191G17	23p	24p
CH261-65O4	24q	25q
CH261-103F4	24p	25q
CH261-127K7	25q	26q
CH261-59C21	25p	26p
CH261-170L23	26q	27q
CH261-186M13	26p	27p
CH261-28L10	27q	28q
CH261-66M16	27p	28p
CH261-64A15	28q	29q
CH261-72A10	28p	29p

## Discussion

Gruiformes is among the avian orders with the least chromosomal information (Furo et al., 2015, 2020). Currently, karyological data obtained by classical cytogenetic methods are available for 30 out of a total of 189 species of Gruifromes. Among these species, only six species (belonging to four out of 33-40 genera) from the family Rallidae were cytogenetically investigated (Garcia et al., 2014; Gill et al., 2020): *Fulica atra,* 2n=92; *Gallinula chloropus,* 2n=78; *Aramides cajaneus*, 2n=78; *G. melanops,* 2n=80; *Porzana albicollis,* 2n=72; *Porphyiro porphyrio,* 2n= 80 (Giannoni and Giannoni, 1983; Hassan, 1998; Nanda et al., 2011; Gunski et al., 2019; Furo *et al*., 2020).

A comparison of chromosome morphology available for this family shows that generally the first six pairs are biarmed, while the remaining macrochromosomes are telocentrics, except in *P. albicollis*, which has a karyotype of only biarmed macrochromosomes (Table 3). Compared to other Gruiformes, which usually follow a chromosome pattern common to each family, there is great diversity in chromosomal morphology in Rallidae species, due to inversions, fusions and fissions, which play an important role in the karyotype evolution within this family (Furo et al., 2015).

**Table 3 -:** Morphological classification of macrochromosomes of five species belonging to the Rallidae family. Legend: M- Metacentric; SM- Submetacentric; T- Telocentric.

	Chromosome morphology										
	Chromosomes										
Species	1	2	3	4	5	6	7	8	9	10	Reference
*Fulica atra*	M	SM	M	M	SM	M	T	T	T	T	Nanda et al., 2011
*Gallinula chloropus*	M	SM	M	M	SM	M	T	T	T	T	Nanda et al., 2011
*Gallinula melanops*	SM	SM	M	M	M	T	T	T	T	T	Gunski et al., 2019
*Porzana albicollis*	M	M	SM	SM	SM	M	M	M	M	M	Giannoni and Giannoni, 1983
*Porphyrio porphyrio*	M	M	M	M	M	SM	SM	A	A	A	Hassan, 1998
*Aramides cajaneus*	SM	SM	T	SM	M	M	T	M	T	T	Furo et al., 2020

Comparative chromosome painting data using *G. gallus* probes in Rallidae species are restricted to three genera: *Fulica*, *Gallinula* and *Aramides* (Nanda et al., 2011; Furo et al., 2020). The chromosome rearrangements found in this group involved chromosomes GGA4, GGA5, GGA6 and GGA7. In *Fulica* and *Gallinula* genera, the fission of GGA4 and GGA5, and fusions between GGA4/GGA5 and GGA6/GGA7 were observed in *F. atra*, *G. chloropus* (Nanda *et al*., 2011) and also in *G. melanops* (2n=80), analyzed herein (Figure 4). Probably, these rearrangements were already present in the common ancestor of Clade Fulica.

Furthermore, it was possible to confirm that each chicken or Zebra finch BAC was conserved as a distinct element in each microchromosome of *G*. *melanops* (Figure 3). Microchromosomes are highly conserved in bird karyotypes, with rearrangements involving these elements detected only is some orders, such as Psittaciformes and Falconiformes (O’Connor et al., 2019). Despite the conservation of microchromosomes in the karyotype of *G*. *melanops*, the increase in diploid number in *F. atra* (2n=92) can be explained by extensive fission of microchromosomes.

According to Sangster et al. (2015), *G. melanops* should be included in the genus *Porphyiro*. However, the chromosome morphology data of *P. porphyrio*, the only species from this genus with a known karyotype, do not show many similarities with the karyotype of *G. melanops*. For example, in *G*. *melanops* the macrochromosomes 1-5 are biarmed and 6-10 are telocentrics, whereas in *P. porphyrio* chromosomes 1-7 are biarmed and 8-10 are acrocentrics (Table 3).

The phylogenetic relationships within the ‘Clade *Fulica*’ (genera *Fulica*, *Gallinula* and *Porzana*), based on mitochondrial DNA (mtDNA), suggest that this group is paraphyletic (Sangster et al., 2015). In the analysis based on mitochondrial and nuclear genes (Cytb*, COI, 16S, FGB-7, RAG-1)*, performed by Garcia et al. (2014), *G. melanops* (2n=80) was recovered as the sister clade to *G. chloropus* (2n=78). However, these species share the same chromosome rearrangements, which could indicate that their common ancestor would contain the fission into GGA4 and GGA5, aside from the association between GGA4/GGA5 and GGA6/GGA7.

**Figure 5 -:**
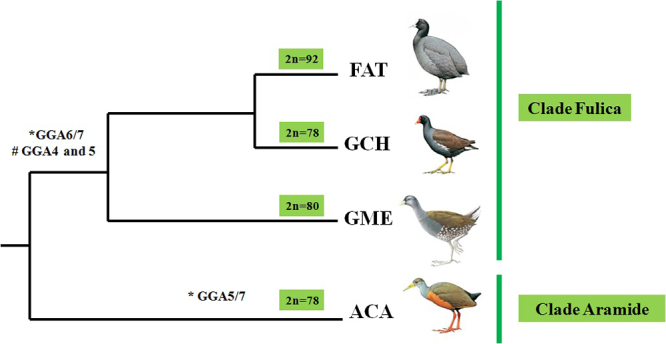
An adaptation of the phylogenetic tree proposed by Garcia-R et al. (2014), plotted with FISH data from *G.gallus* probes (Legend: FAT- *Fulica atra*; GCH- *Gallinula chloropus*; ACA- *Aramides cajaneus*; GME- *Gallinula melanops*). (*) correspond fusion and (#) fission.

Furthermore, other phylogenetic analyses using mtDNA recovered *G. melanops* in a more basal position within Clade *Fulica* (species of genera *Fulica* and *Gallinula*) (Garcia et al., 2014; Sangster et al., 2015), consistent with the chromosome painting data that indicate the karyotypic similarity between *G. melanops* and *G. chloropus* (Figure 5)*.*

Additionally, the clade *Aramides* would be sister group to clade *Fulica,* despite the species *A. cajaneus* (Clade *Aramides*) not showing the fission into GGA4 and GGA5, or the fusions between GGA4/GGA5 and GGA6/GGA7 (Furo et al., 2020). Thus, the last common ancestor of these clades would have a karyotype similar to the putative avian ancestral karyotype (Furo *et al*., 2020).

In conclusion, the comparative chromosome painting reveals that *G. melanops* has a similar karyotype to *G. chloropus* and does not support the separation of these species into different genera. They are supported as sister species. Additionally, as in most birds studied so far, the microchromosomes are conserved as distinct pairs and do not take part in interchromosomal rearrangements (fusions or fissions). The results illustrate the value of comparative chromosome painting and BAC mapping in phylogenetic studies.
